# Effects of Adjuvant Medications on A1C, Body Mass Index, and Insulin Requirements among Patients with Type 1 Diabetes

**DOI:** 10.3390/pharmacy10040097

**Published:** 2022-08-08

**Authors:** Armando Silva Almodóvar, Jonathan Clevenger, Milap C. Nahata

**Affiliations:** 1 Institute of Therapeutic Innovations and Outcomes (ITIO), College of Pharmacy, Ohio State University, Columbus, OH 43210, USA; 2 College of Medicine, Ohio State University, Columbus, OH 43210, USA

**Keywords:** type 1 diabetes, adjuvant medications, insulin, body mass index, glycated hemoglobin, off-label

## Abstract

Randomized controlled trials have demonstrated that noninsulin medications used to treat type 2 diabetes can improve health outcomes among patients with type 1 diabetes (T1D). This study assessed the effects of adjuvant diabetes medications on glycated hemoglobin (A1C), body mass index (BMI), or total daily insulin (TDI) among patients with T1D in a real-world setting. This was an analysis of the T1D Exchange Clinic Registry, using the study periods of 2010–2012, 2015–2016, and 2016–2017, to assess differences in A1C, BMI, and TDI between patients with and without adjuvant medications. The relationships between characteristics and A1C in 2015–2016 and 2016–2017 were determined. Analysis included 517 patients in the adjuvant medication cohort and 4968 in the insulin-only cohort. No significant improvement in A1C was observed. A significant difference in BMI and TDI between the insulin-only (median BMI: 25.5, 26.2, 26.4 and median TDI: 45, 44 units) and adjuvant medication cohorts (median BMI: 29.8, 30.5, 30.5 and median TDI: 51, 52 units) (*p* < 0.001) was observed. Patients with a continuous glucose monitor (CGM), higher education level, higher annual income, and older age were associated with lower A1C (*p* ≤ 0.001). Higher BMI and self-description as African American/Black were associated with higher A1C (*p* ≤ 0.01). Insulin pump use was associated with lower A1C (*p* < 0.01) in 2015–2016. Patients who used adjuvant medications did not demonstrate significant improvement in disease control. These data suggest that findings from well-designed research studies may not be consistently reproducible in real-world settings, due to patient-specific factors.

## 1. Introduction

Type 1 diabetes (T1D) is an autoimmune disease that leads to the elimination of pancreatic beta cells, resulting in complete insulin deficiency [1]. Insulin is necessary for the effective uptake of plasma glucose in muscle and adipose cells and storage of glucose within the liver [1]. Treatment with insulin is necessary to prevent the development of complications associated with high levels of plasma glucose, such as diabetic ketoacidosis, cardiovascular disease, and chronic kidney disease [1]. It was estimated that, worldwide, 9 million people lived with T1D in 2017 [2]. T1D is estimated to cost these patients and payers approximately USD 800 billion over a lifetime [3]. Glycemic control among individuals with T1D is critical to reduce related morbidity and mortality and, consequently, overall cost of care [4]. However, only approximately 20% of adults with T1D achieved adequate glycemic control, as established by the American Diabetes Association [5].

Further complicating the treatment of patients with T1D is the high prevalence of obesity [6]. Obesity can lead to an increased risk for insulin resistance, inflammation, dyslipidemia, and metabolic syndrome, complications commonly seen among patients with type 2 diabetes (T2D) [6]. The presence of these contributing factors can increase an individual’s insulin needs and overall medication burden, potentially aggravating effective management of T1D. Noninsulin antidiabetic medications used to treat T2D have been used in randomized controlled trials (RCTs) among patients with T1D to improve glycated hemoglobin (A1C), achieve weight loss, and reduce cardiovascular risk [7,8,9,10,11]. Currently, the only noninsulin analog with approval by the Food and Drug Administration (FDA) to treat T1D is pramlintide, an amylin analog [7]. Pramlintide may reduce postprandial glucose absorption by delaying gastric emptying, reducing glucose absorption, increasing satiety, and inhibiting secretion of glucagon [7]. Although effective, this medication may not be suitable for some patients due to the requirements of an additional injection at each meal [7]. Further, nausea and vomiting associated with pramlintide may limit its use [7].

In addition to pramlintide, numerous other medications commonly used for the treatment of T2D are used in patients with T1D [12]. Also known as adjuvant medications, these medications are used adjunctively among patients with T1D to decrease A1C and reduce total daily insulin (TDI) or body mass index (BMI) [7,8,9,10,11]. To date, no studies have examined the overall use of these medications and their potential impact on outcomes under real-world conditions among patients with T1D. Two studies have examined the use of sodium–glucose cotransporter-2 (SGLT-2) inhibitors in a real-world population; however, one study did not record use of continuous glucose monitors (CGM), limiting the application of the results to clinical practice [13,14]. The objective of this study was to examine the effectiveness of insulin alone versus insulin plus adjuvant medication use on A1C, BMI and TDI among patients with T1D.

## 2. Materials and Methods

This was a retrospective observational cohort analysis of de-identified datasets obtained from the T1D Exchange Clinic Registry. The T1D Exchange Clinic Registry collected information from participants from 67 endocrinology centers in the United Sates (US) who elected to enroll and complete a questionnaire with demographic information and medical history. Data were obtained from medical records of patients. Patients who qualified for follow-up studies were identified and contacted using the information provided during initial screening. All T1D Exchange Clinic Registry study materials had been reviewed and approved by JAEB IRB (Tampa, FL, USA). The Ohio State University Institutional Review Board determined that this study was exempt from review.

Investigators obtained three datasets for the following clinical exam dates: 1 September 2010 to 1 August 2012 (2010–2012 study period), 30 April 2015 to 31 July 2016 (2015–2016 study period), and 1 May 2016 to 31 July 2017 (2016–2017 study period). A patient identification number was utilized to link relevant data pertaining to each patient across the datasets. Initially, all patients were included. Patients without a medication record or self-reported as pregnant in the 2015–2016 and 2016–2017 datasets were excluded. Patients in the 2015–2016 study period who were 18 years of age or younger or 75 years of age or older were also excluded. Medications were assessed in the 2015–2016 and 2016–2017 datasets. Patients without use of a medication to treat type 2 diabetes in the 2015–2016 and 2016–2017 datasets were identified as insulin-only users. Patients with insulin and additional medications to treat type 2 diabetes in the 2015–2016 and 2016–2017 study periods were identified as adjuvant medication users. Medications included in the analysis were metformin, SGLT2 inhibitors, glucagon-like peptide-1 (GLP-1) receptor agonists, pramlintide, sulfonylureas, bile acid sequestrants, meglitinides, bromocriptine, and colesevelam, due to being used in >10 patients.

### Statistical Analysis

Data were coded and organized using Microsoft Excel (2016 MSO, Redmond, WA, USA) and IBM SPSS software (v25.0, IBM Corp., Armonk, NY, USA). Counts, percentages, medians and interquartile ranges were used to describe the population. Demographic data for patients was collected from the 2016–2017 dataset. Chi-squared tests and sign tests were used to assess differences between the insulin-only and the adjuvant medication cohorts, using the 2016–2017 study period. To assess if the use of adjuvant medications was associated with improvement in BMI or A1C, these outcome variables were collected from the 2010–2012, 2015–2016, and 2016–2017 study periods. The TDI outcome variable was only collected during the 2015–2016 and 2016–2017 datasets, due to differences in how data was recorded in the 2010–2012 study period.

A two-way mixed repeated measures ANOVA assessed the differences in A1C and BMI, between the insulin-only and the adjuvant medication cohorts, using the 2010–2012, 2015–2016, 2016–2017 study periods. TDI values were compared between the 2015–2016 and 2016–2017 study periods. A1C and BMI were transformed using natural log. Outliers were defined as having a studentized residual less than −3 or greater than 3. Q-Q plots determined the studentized residuals for normality. A sensitivity analysis was performed, omitting outliers. Mauchly’s test for sphericity was violated during the execution of the mixed ANOVA analyses. Thus, within and between subjects’ effects were adjusted using the Huynh–Feldt correction. Given the assumption for Box’s test of equality of variance was violated and within subject interactions were detected, Friedman tests were also used to assess for differences in A1C and BMI between 2010–2012, 2015–2016, and 2016–2017 separately, among insulin plus adjuvant medication users versus the insulin-only cohort. Sign tests assessed differences in TDI between the 2015–2016 and 2016–2017 study periods in each cohort individually.

Linear regression assessed the relationship between unique patient characteristics with A1C in the 2015–2016, and 2016–2017 study periods. The A1C was transformed using natural log for the linear regression analysis. BMI was converted to categories for the regression analysis [15]. The results of the regression analysis were reported as standardized and unstandardized beta coefficients and *p* values. Variables included in the regression were self-reported age, BMI, Black/African American, self-reported as other ethnicity, gender, education level, income level, use of adjuvant medication, use of CGM, and use of an insulin pump. A two-tailed, a priori alpha level of 0.05 defined statistical significance. Bonferroni corrections were made for post-hoc testing of the Friedman test.

## 3. Results

A total of 33,666 unique patients were initially considered for inclusion into the study. After the application of exclusion criteria, there were 517 patients in the adjuvant medication cohort and 4968 patients in the insulin-only cohort. The majority of patients included for analysis were female (3000, 55%), did not smoke (5063, 92%), did not use CGM devices (3508, 64%), did not use an insulin pump (3566, 65%), reported earning more than USD 50,000 per year (1559, 55%%), self-described as White non-Hispanic (4870, 89%), received a degree from college (3315, 60%), had an A1C greater than 7.5%, (2707, 49%), and a BMI greater than 25kg/m^2^ (3063, 56%). Patients had a median age of 44 years and used a median of 45 insulin units per day.

Among patients in the adjuvant medication cohort, there were 233 on metformin, 107 on SGLT2 inhibitors, six on DPP4 inhibitors, 117 on GLP1 agonists, eight on thiazolidinediones, four on sulfonylureas, 79 on pramlintide, 30 on a bile acid sequestrant, and none on meglitinides or bromocriptine. In this cohort there were 53 patients on two adjuvant medications, four patients on three adjuvant medications, and two individuals on four adjuvant medications.

Chi-squared and sign tests revealed that patients in the adjuvant medication cohort, when compared to the insulin-only cohort, were more likely to be female (*p* < 0.001), less likely to smoke (*p* < 0.003), more likely to use a CGM device (*p* < 0.002), older (*p* < 0.001), with a higher BMI (*p* < 0.001), and utilizing more insulin units per day (*p* < 0.001), when compared to patients who only used insulin (Table 1). Complete demographic information can be found in Table 1.

### 3.1. A1C

#### 3.1.1. A1C Mixed Repeated Measures ANOVA Analysis (2010–2012, 2015–2016, 2016–2017)

**Figure 1:**
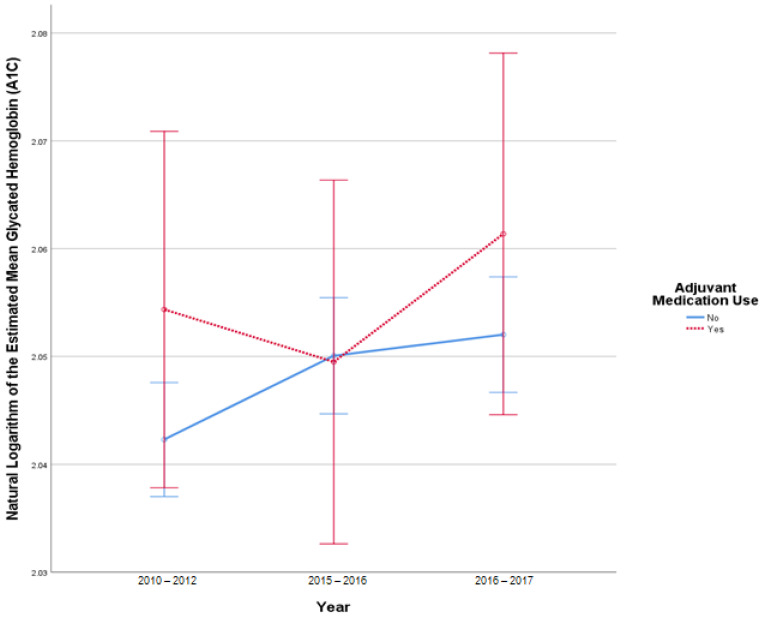
Results from mixed repeated measures ANOVA comparing the glycated hemoglobin (A1C) of individuals in the control cohort versus patients with adjuvant medication use between 2010 and 2017. Error bars reflect 95% confidence interval. Figure illustrates changes in A1C among patients with and without adjuvant medication use over time.

This analysis included 3615 patients in the insulin-only cohort and 369 patients in the adjuvant medication cohort, as these patients had a reported A1C in all 3 datasets. There were 41 outliers in 2010–2012, 46 outliers in 2015–2016, and 45 in 2016–2017. There was slight deviation of normality at the tail end of the Q-Q plots of the studentized residuals. There was no homogeneity of covariances as assessed by Box’s test of equality of covariance matrices (*p* < 0.001). Mauchly’s test for sphericity indicated that the assumption of sphericity was violated for the two-way interaction, x^2^ (2) = 829.149, *p* < 0.001. There was no statistically significant interaction between time and use of adjuvant medications, F (1.685, 6707.808) = 3.276, *p* = 0.17, partial η^2^ < 0.001. There was a statistically significant difference in mean A1C at the different date ranges, F (1.685, 6707.808) = 3.276, *p* = 0.046, partial η^2^ = 0.001. There was no statistically significant difference in mean A1C between the adjuvant medications versus insulin-only group F (1, 3982) = 0.755, *p* = 0.39, partial η^2^ < 0.001. Figure 1 illustrates the change in A1C over time.

#### 3.1.2. A1C Mixed Repeated Measures ANOVA Sensitivity Analysis (2010–2012, 2015–2016, 2016–2017)

**Figure A1:**
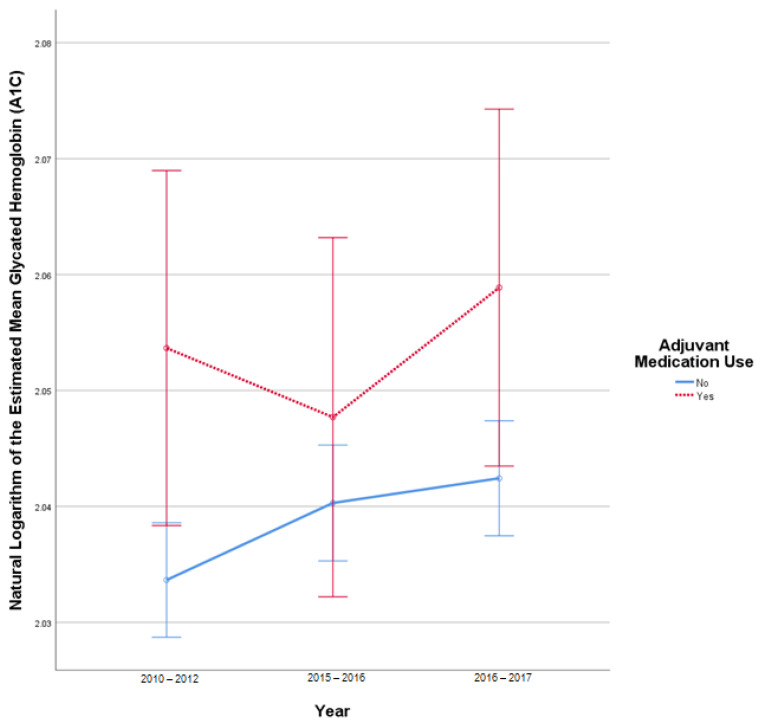
Results from mixed repeated measures ANOVA comparing the glycated hemoglobin (A1C) of individuals in the control cohort versus patients with adjuvant medication use between 2010 and 2017. Error bars reflect 95% confidence interval. Outliers omitted. Figure illustrates changes in A1C among patients with and without adjuvant medication use over time.

This analysis included 3520 patients in the insulin-only cohort and 366 patients with adjuvant medication use after omitting outliers. Q-Q plots of the studentized residuals reflected normality. Homogeneity of covariances was confirmed, as assessed by Box’s test of equality of covariance matrices (*p* = 0.069). Mauchly’s test for sphericity indicated that the assumption of sphericity was violated for the two-way interaction, x^2^ (2) = 692.505, *p* < 0.001. There was no statistically significant interaction between time and the use of adjuvant medications, F (1.72, 6681.696) = 1.947, *p* = 0.15, partial η^2^ = 0.001. There was no statistically significant difference in mean A1C at the different study periods, F (1.720, 6681.696) = 2.866, *p* = 0.07, partial η^2^ = 0.001. There was a statistically significant difference in mean A1C between the use of adjuvant medications and the insulin-only group F (1, 3884) = 3.983, *p* = 0.046, partial η^2^ = 0.001. Figure A1 illustrates the change in A1C over time without outliers.

#### 3.1.3. Assessing Change in A1C over Time in Each Cohort Individually (2010–2012, 2015–2016, 2016–2017)

A Freidman test found a statistically significant difference between the three time points in A1C, in the insulin-only (x^2^ (2) = 14.456, *p* = 0.001) and the adjuvant medication cohort (x^2^ (2) = 8.229, *p* = 0.016). Sign tests with Bonferroni corrections were used for post-hoc comparisons. In the insulin-only cohort, there was a statistically significant difference in A1C between 2010–2012 (median = 7.6%) and 2016–2017 (median = 7.7%, *p* < 0.001). In the adjuvant medication cohort, there was a statistically significant difference in A1C between 2015–2016 (median = 7.6%) and 2016–2017 (median = 7.8%, *p* = 0.004). Individual assessments of each adjuvant medication cohort found no statistically significant difference in A1C at any time point (Table 2).

### 3.2. BMI

#### 3.2.1. BMI Mixed Repeated Measures ANOVA Analysis (2010–2012, 2015–2016, 2016–2017)

**Figure 2:**
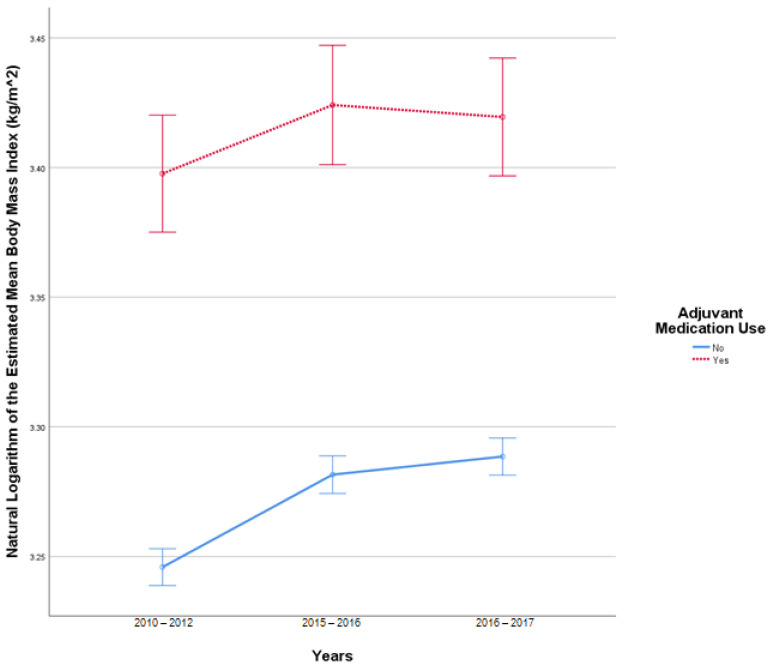
Results from mixed repeated measures ANOVA comparing the body mass index (BMI) of individuals in the control cohort versus patients with adjuvant medication use between 2010 and 2017. Error bars reflect 95% confidence interval. Figure illustrates changes in BMI among patients with and without adjuvant medication use over time.

This analysis included 2519 patients in the insulin-only cohort and 250 patients with adjuvant medication, use as these patients had BMI reported at all three time points. In this sample, there were 17 outliers in 2010–2012, 20 outliers in 2015–2016, and 19 outliers in 2016–2017. Q-Q plots of the studentized residuals showed slight deviation from normality at the tail ends. There was no homogeneity of covariances, as assessed by Box’s test of equality of covariance matrices (*p* < 0.001). Mauchly’s test for sphericity indicated that the assumption of sphericity was violated for the two-way interaction, x^2^ (2) = 771.476, *p* < 0.001. There was a statistically significant interaction between time and the use of adjuvant medications F (1.610, 4454.524) = 3.821, *p* = 0.031 partial η^2^ = 0.001. There was a statistically significant difference in BMI at different study periods F (1.610, 4454.524) = 47.097, *p* < 0.001, partial η^2^ = 0.017. There was a statistically significant difference in mean BMI between the adjuvant medication cohort and the insulin-only cohort F (1, 2767) = 155.396, *p* < 0.001, partial η^2^ = 0.053. Figure 2 illustrates the change in BMI over time.

#### 3.2.2. BMI Mixed Repeated Measures ANOVA Sensitivity Analysis (2010–2012, 2015–2016, 2016–2017)

**Figure A2:**
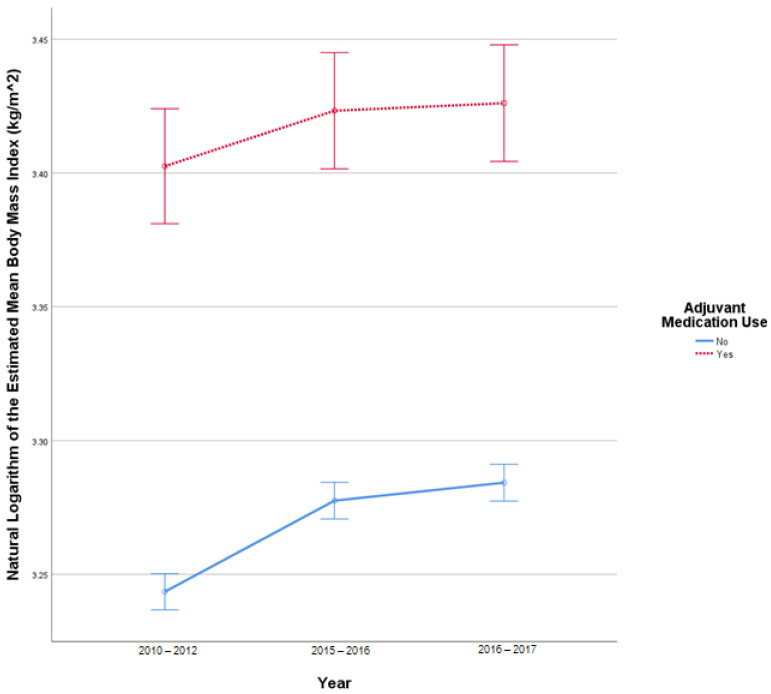
Results from mixed repeated measures ANOVA comparing the body mass index (BMI) of individuals in the control cohort versus patients with adjuvant medication use between 2010 and 2017. Error bars reflect 95% confidence interval. Outliers omitted. Figure illustrates changes in BMI among patients with and without adjuvant medication use over time.

After omitting outliers, there were 2484 patients in the insulin-only group and 247 patients in the adjuvant medication cohort. Q-Q plots of the studentized residuals reflected normality. There was no homogeneity of covariances, as assessed by Box’s test of equality of covariance matrices (*p* < 0.001). There was a statistically significant interaction between study periods and use of adjuvant medications F (1.504, 4105.512) = 3.901, *p* = 0.03, partial η^2^ =0.001. There was also a statistically significant difference in BMI at the different study periods, F (1.504, 4105.512) = 57.701, *p* < 0.001, partial η^2^ =0.021. A statistically significant difference was observed in BMI between the adjuvant medication and insulin-only cohorts F (1, 2729) = 184.042, *p* < 0.001, partial η^2^ = 0.063. Figure A2 illustrates the change in BMI over time without the outliers.

#### 3.2.3. Assessing Change in BMI over Time in Each Cohort Individually (2010–2012, 2015–2016, 2016–2017)

A Freidman test showed a statistically significant difference between the three study periods in BMI, in the insulin-only (x^2^ (2) = 367.659, *p* < 0.001) and the adjuvant medication cohort (x^2^ (2) = 8.504, *p* = 0.014). Sign tests with Bonferroni corrections were used for post-hoc comparisons. In the insulin-only cohort, there was a statistically significant difference in BMI between 2010–2012 (median = 25.5 kg/m^2^), 2015–2016 (median = 26.2 kg/m^2^), and 2016–2017(median = 26.4 kg/m^2^, *p* < 0.001). In the adjuvant medication cohort, there was a statistically significant difference in BMI between 2010–2012 (median = 29.8 kg/m^2^) and 2015–2016 (median = 30.5 kg/m^2^, *p* = 0.004) and between 2015–2016 (median = 30.5 kg/m^2^) and 2016–2017 (median = 30.5 kg/m^2^, *p* < 0.001) (Table 2).

### 3.3. TDI

#### 3.3.1. TDI Mixed Repeated Measures ANOVA Analysis (2015–2016, 2016–2017)

**Figure 3:**
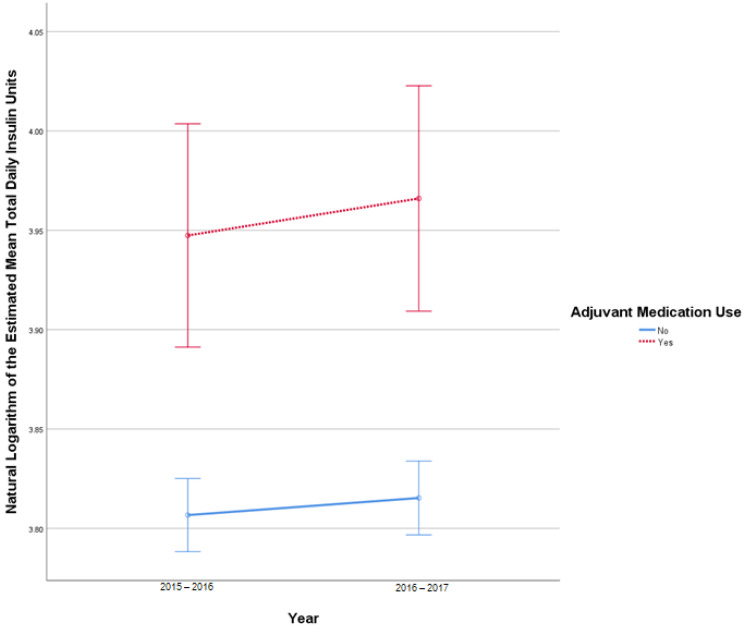
Results from mixed repeated measures ANOVA comparing the total daily insulin (TDI) units of individuals in the control cohort versus patients with adjuvant medication use between 2010 and 2017. Error bars reflect 95% confidence interval. Figure illustrates changes in TDI among patients with and without adjuvant medication use over time.

This analysis included 2681 patients in the insulin-only cohort and 287 patients in the adjuvant medication cohort. There were 21 TDI outliers identified in 2015–2016 and 24 TDI outlier values in 2016–2017. Deviation from normality was identified at the tail end of Q-Q plots. Homogeneity of covariances was confirmed by Box’s test of equality of covariance matrices (*p* = 0.150). There was no statistically significant interaction between the use of adjuvant medications and time on TDI, F (1, 2966) = 0.421, *p* = 0.52. There was no significant effect of time on TDI F (1, 2966) = 3.127, *p* = 0.08, partial η^2^ = 0.001. There was a statistically significant difference in average TDI between the adjuvant medication and the insulin-only group, F (1, 2966) = 24.702, *p* < 0.001, partial η^2^ = 0.008. Figure 3 describes the change in TDI over time.

#### 3.3.2. TDI Mixed Repeated Measures ANOVA Sensitivity Analysis (2015–2016, 2016–2017)

**Figure A3:**
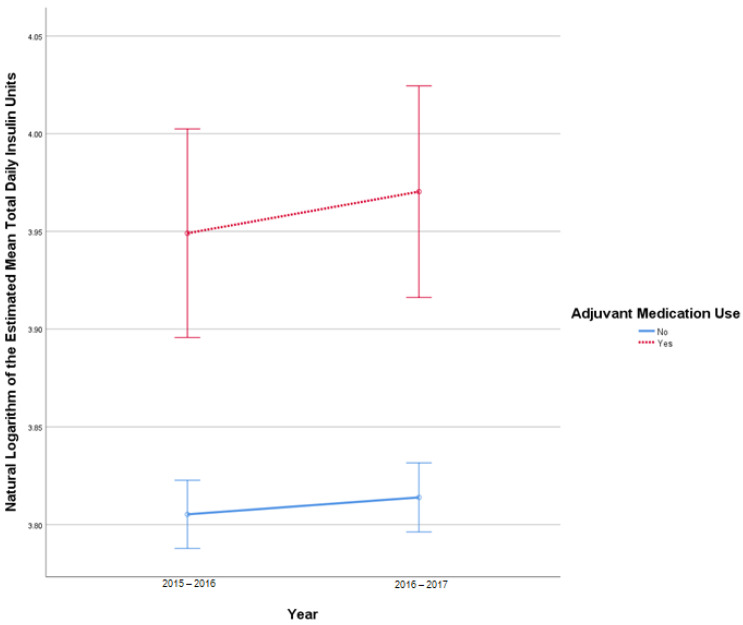
Results from mixed repeated measures ANOVA comparing the total daily insulin units (TDI) of individuals in the control cohort versus patients with adjuvant medication use between 2010 and 2017. Error bars reflect 95% confidence interval. Outliers omitted. Figure illustrates changes in TDI among patients with and without adjuvant medication use over time.

This analysis included 2655 patients in the insulin-only cohort and 283 with adjuvant medication use. Q-Q plots of the studentized residuals reflected normality (*p* = 0.179). There was no statistically significant interaction between the use of adjuvant medications and time on TDI, F (1, 2936) = 0.744, *p* = 0.39, partial η^2^ < 0.001. There was a statistically significant effect of time on TDI between 2015 and2017 F (1, 2936) = 4.195, *p* = 0.04, partial η^2^ = 0.001. There was a statistically significant difference in average TDI between the adjuvant medication and the insulin-only group, F (1, 2936) = 28.933, *p* < 0.001, partial η^2^ = 0.010. Figure A3 illustrates the change in TDI over time without the outliers.

#### 3.3.3. Assessing Change in TDI over Time in Each Cohort Individually (2015–2016, 2016–2017)

Sign tests found a statistically significant difference in TDI in the insulin-only group between 2015–2016 (median 45 units) and 2016–2017 (median 44 units, *p* < 0.001). No significant difference between 2015–2016 and 2016–2017 was found in the adjuvant medication cohort (*p* = 0.95) (Table 2).

### 3.4. Multivariate Linear Regressions (2015–2017)

Two multivariate regression models were used to determine the relationship between unique patient characteristics with A1C using data from the 2015–2016 and 2016–2017 study periods. Regressions using data from 2015–2016 (F (10, 3417) = 46.156, *p* < 0.001, R^2^ = 0.12) and 2016–2017 (F (10, 3440) = 54.581, *p* < 0.001, R^2^ = 0.13) each significantly predicted A1C. The use of a CGM, higher education level, higher annual income, and older age had a statistically significant association with lower A1C (*p* ≤ 0.001) in both datasets. Self-description as African American/Black and higher BMI had a direct significant relationship with higher A1C in both datasets (*p* ≤ 0.01). Use of an insulin pump was associated with lower A1C (*p* < 0.01), in the 2015–2016 dataset. This relationship was not observed in the 2016–2017 study period. Regression coefficients can be found in Table 3.

## 4. Discussion

The effective management of glucose levels among patients with T1D remains a significant challenge. A previous study found that approximately one in five adults with T1D achieve A1C levels below 7% [5]. A variety of antidiabetic medications approved for T2D have been used to improve disease control among patients with T1D receiving insulin therapy. To our knowledge, this is the first study to assess the effectiveness of adjuvant medications (in addition to insulin) over a 7-year period on A1C, BMI and TDI among adult patients with T1D in a real-world setting. We found no significant improvement in A1C, BMI, and TDI among individuals who used adjuvant medications from 2015 to 2017, in comparison to previous measurements of disease control between 2010 and 2017. Additionally, we found the most important unique patient characteristics associated with lower A1C were use of a continuous glucose monitor (CGM), older age, higher annual income, and greater education level. Higher BMI and being self-described as African American/Black were associated with higher A1C. These findings demonstrated that results from clinical trials may not be consistently replicated in real-world populations, due to important specific patient related factors and concomitant treatments.

In this study, patients with adjuvant medication use presented with no significant difference in A1C between 2010 and 2017, when compared to an insulin-only cohort. Additionally, the data suggested that A1C modestly increased over time among both adjuvant medication users and the insulin-only cohort. BMI significantly increased over time in both the adjuvant medication and control cohorts in this time period. TDI increased significantly in the insulin-only cohort from 2015 to 2017. It is important to note no significant difference in A1C, BMI, and TDI values were observed when adjuvant medication cohorts were examined for individual drugs, except for the metformin cohort that was associated with a significant increase in BMI.

The findings from this study may differ from those reported in randomized controlled clinical trials for medication classes such as SLGT-2 inhibitors, amylin analogs, GLP-1 receptor analogs, and DPP4 inhibitors, which have demonstrated clinically significant and positive results on reducing body weight, total daily insulin needs, and A1C among patients with type 1 diabetes [7,8,9,10,11]. This may have occurred because clinical trials typically followed patients only for one year, and one metformin trial assessed patients for three years [8], while this study utilized data from patients over seven years. Furthermore, patients in real-world settings may not benefit from the stringent standard of care, frequent monitoring or follow-up and free access to important medications and interventions provided in randomized clinical trials. An additional study of the T1D Exchange data examining SGLT2 inhibitors found that these medications were not associated with improvements in A1C, similar to our findings among patients receiving SGLT2 inhibitors [14]. These results differ from a retrospective review of patients with T1D, which found that SGLT2 inhibitors were associated with improvements in A1C, TDI, and BMI after a year of treatment [13]. However, it is important to note that these patients received care at academic medical centers participating in clinical trials examining the effects of SGLT2 inhibitors among patients with T1D [12].

Finally, given that the brand medications were used off-label in this study, medication adherence may have been compromised due to various factors including costs, variations in insurance coverage, and health literacy. As an example, one study found that 17% of patients with diabetes were considered non-adherent to their medications, and this increased with the presence of insulin prescriptions and obesity [16]. Clinical outcomes would not be achieved as expected if patients are not utilizing their medications as prescribed. Additionally, differences in inflammation and drug metabolism caused by worsening diabetes control could have also contributed to a reduced response among patients utilizing adjuvant medications [17,18,19,20].

The findings from this study importantly emphasize targeted disease management approaches. Older age, higher annual income, the use of CGM, and higher education level were significantly associated with lower A1C. Consistent with previous studies, CGM devices were associated with lower A1C, as they provide patients with consistent access to glucose data that can allow the identification of glucose trends, assist in the optimization of therapies, and alarm patients to significant hyper- and hypoglycemia [21]. Older patients are likely to receive more frequent contact with health care providers, resulting in greater exposure to relevant health information and reinforcement of important lifestyle interventions for patients with type 1 diabetes [22,23]. Furthermore, older patients are more likely to be adherent to their medication regimens, potentially related to a greater understanding of the importance of control of chronic health conditions [23]. Patients with higher income levels may have greater access to health care services [24,25], have higher health literacy [26], more easily afford the high cost of insulin products resulting in greater adherence [27], and have greater access to healthy foods [28], when compared to patients with lower incomes. Finally, patients with higher education levels may have higher health literacy [26] and be more likely to adhere to their pharmacological and disease management strategies [27].

Also identified in this study was a positive association between higher BMI and being self-described as African American/Black with higher A1C. The relationship between BMI and A1C may signal increased insulin resistance [29] in this population or serve as a surrogate marker for nonadherence to lifestyle therapies. The relationship between being self-described as African American/Black and having higher A1C was consistent with other studies that found that these patients were more likely to have lower health literacy [26] and be less adherent to their therapeutic regimen [30].

There were several limitations to consider in this study. This was a retrospective analysis of data from patients with type 1 diabetes in a publicly available database. Medication adherence data were not available in this database. Patients in this cohort were predominately White non-Hispanic; thus, results may not be applicable to other ethnicities. Chronic disease burden, efficacy data, and reasons for prescribing adjuvant medications were not available in the database. Finally, although this was the first longitudinal study to determine the effects of adjuvant medications among patients receiving insulin for type 1 diabetes over a 7-year period in the real-world settings documented in a national database, certain small subgroups of patients may have been affected by type 2 error.

## 5. Conclusions

Patients with type 1 diabetes who used adjuvant medications with insulin did not have significant improvement in A1C between 2010–2012, 2015–2016, and 2016–2017, when compared to an insulin-only cohort. BMI significantly increased in both cohorts. Total daily insulin use modestly decreased in the insulin-only cohort from 2015–2016 to 2016–2017. Use of adjuvant medications among patients with type 1 diabetes was not associated with improvements in A1C, BMI, or TDI in this study. Older age, higher income, greater education level, and use of a CGM was associated with lower A1C. Higher BMI and patients who self-described as African American/Black were associated with higher A1C. These data suggest that the findings from randomized controlled studies may not be consistently reproduced in the real-world settings over an extended period. This is the first study to examine the effects of various adjuvant medications among patients with type 1 diabetes in a real-world setting. Future studies evaluating the impact of adherence to medications among patients with type 1 diabetes may illustrate the effects and benefits of these medications. Given the results of the current study, healthcare providers should consider targeted lifestyle and disease management approaches and the use of insulin pumps and CGM devices to improve A1C, BMI, or TDI among patients with Type 1 diabetes.

## Figures and Tables

**Table 1:** Characteristics of patients with Type 1 diabetes in the year 2016–2017.

Characteristics	Insulin-Only User (*n* = 4968)	Insulin + Adjuvant Medication Use (*n* = 517)	*p* Value	Total Cohort (N: 5485)
	(Median (25th–75th interquartile range)			
Age (years)	43.0 (28.0–58.0)	47.0 (36.0–56.0)	0.001	44.0 (28.0–57.0)
Body Mass Index, (kg/m^2^)	26.7 (23.7–30.2)	30.4 (26.6–35.2)	<0.001	26.9 (23.9–30.7)
Diabetes Duration (years)	22.9 (14.3–35.6)	22.3 (14.3–34.0)	0.20	22.9 (14.3–35.4)
A1C (%)	7.6 (6.9–8.5)	7.7 (7.1–8.6)	0.23	7.6 (7.0–8.5)
Total Daily Insulin (Units)	45.0 (33.0–61.0)	53.0 (37.0–74.0)	<0.001	45.0 (33.0–62.0)
	N (%)	N (%)		
Sex			<0.001	
Male	2274 (46%)	194 (38%)		2468 (45)
Female	2679 (54%)	321 (62%)		3000 (55)
Unknown	15 (0%)	2 (0%)		17 (0)
Ethnicity			0.78	
White non-Hispanic	4406 (89%)	464 (90%)		4870 (89)
African American/Black	214 (4%)	19 (4%)		233 (4)
Other Minority	311 (6%)	32 (6%)		343 (6)
Hispanic or Latino	170 (3%)	17 (3%)	-	187 (3)
Native Hawaiian/Other Pacific Islander	3 (0%)	0 (0)		3 (0)
Asian	46 (1%)	4 (1%)		50 (1)
American Indian/Alaskan Native	10 (0%)	4 (1%)		14 (0)
More than one race	82 (2%)	7 (1%)		89 (2)
Unknown	37 (1%)	2 (0%)		39 (1)
Education Level			0.33	
Less than High School graduate	123 (3%)	14 (3%)		137 (3)
High School graduate/diploma/GED	560 (11%)	49 (10%)		609 (11)
Some College but no degree	950 (19%)	90 (17%)		1040 (19)
Associate Degree	457 (9%)	59 (11%)		516 (9)
Bachelor’s Degree	1482 (30%)	150 (29%)		1632 (30)
Master’s Degree	750 (15%)	94 (18%)		844 (15)
Professional Degree	180 (4%)	21 (4%)		201 (4)
Doctorate Degree	108 (2%)	14 (3%)		122 (2)
Unknown	358 (7%)	26 (5%)		384 (7)
Annual Income (USD)			0.96	
Less than 25,000	316 (6%)	31 (6%)		347 (6)
25,000–34,999	236 (5%)	25 (5%)		261 (5)
35,000–49,999	416 (8%)	43 (8%)		459 (8)
50,000–74,999	651 (13%)	73 (14%)		724 (13)
75,000–99,999	653 (13%)	72 (14%)		725 (13)
$100,000 or more	1396 (28%)	163 (32%)		1559 (28)
Unknown	1300 (26%)	110 (21%)		1410 (26)
Smoking Status			0.003	
Yes	271 (6)	12 (2%)		283 (5)
No	4566 (92)	497 (96%)		5063 (92)
Unknown	131 (3)	8 (2%)		139 (3)
Use of Continuous Glucose Monitor (CGM)			0.002	
Yes	1651 (33%)	208 (40%)		1859 (34)
No	3207 (65%)	301 (58%)		3508 (64)
Unknown	110 (2%)	8 (2%)		118 (2)
Use on Insulin Pump			0.23	
Yes	3217 (65%)	349 (68%)		1901 (35)
No	1734 (35%)	167 (32%)		3566 (65)
Unknown	17 (0%)	1 (0%)		18 (0)

Data Collected from T1D Exchange Annual Case Report Form.

**Table 2:** Results from Friedman and sign tests assessing change in A1C, body mass index (BMI), and total daily insulin units (TDI). Outcomes are reported as median (25th–75th interquartile range).

	Medication	2010–2012	2015–2016	2016–2017	*p* Value
A1C	Insulin-only (*n* = 3615)	7.6 (6.9–8.4)	7.7 (7.0–8.5)	7.7 (7.0–8.5)	0.001 ^b^
	Insulin + adjuvant medication use (*n* = 369)	7.7 (7.1–8.5)	7.6 (7.1–8.4)	7.8 (7.2–8.6)	0.02 ^c^
	Metformin (*n* = 151)	7.7 (7.2–8.5)	7.7 (7.1–8.7)	7.9 (7.4–8.7)	0.13
	SGLT2 (*n* = 84)	8.0 (7.4–8.9)	7.9 (7.3–8.6)	8.0 (7.4–8.7)	0.37
	GLP1 (*n* = 85)	7.6 (7.1–8.5)	7.5 (7.0–8.2)	7.5 (7.0–8.3)	0.42
	Pramlintide (*n* = 59)	7.3 (6.8–8.2)	7.3 (6.8–8.0)	7.5 (6.8–8.3)	0.66
	Colesevelam (*n* = 23)	7.8 (7.0–8.6)	7.7 (7.2–8.4)	7.7 (7.1–8.8)	0.16
	Insulin-only (*n* = 2519)	25.5 (22.7–28.8)	26.2 (23.5–29.7)	26.4 (23.6–30.0)	<0.001 ^abc^
BMI	Insulin + adjuvant medication use (*n* = 250)	29.8 (26.1–33.9)	30.5 (26.7–35.6)	30.5 (26.9–35.4)	0.01 ^ac^
	Metformin (*n* = 109)	30.9 (27.2–34.1)	31.6 (27.9–35.9)	31.6 (28.4–35.9)	0.01 ^ac^
	SGLT2 (*n* = 67)	30.0 (25.9–33.5)	30.6 (27.1–34.1)	30.3 (27.1–33.7)	0.48
	GLP1 agonist (*n* = 46)	29.7 (25.6–34.8)	30.0 (25.7–34.5)	30.2 (25.2–33.9)	0.25
	Pramlintide (*n* = 39)	29.1 (26.3–36.0)	29.6 (27.4–36.9)	30.5 (27.5–35.8)	0.80
	Colesevelam (*n* = 17)	31.1 (26.6–33.2)	30.5 (27.3–35.1)	29.9 (26.5–36.7)	0.84
	Insulin-only (*n* = 2689)	-	45.0 (33.0–60.0)	44.0 (33.0–61.0)	<0.001
	Insulin + adjuvant medication use (*n* = 287)	-	51.0 (36.0–73.0)	52.0 (38.0–73.0)	0.95
TDI	Metformin (*n* = 127)	-	60.0 (44.0–84.0)	59.0 (44.0–81.0)	1.0
	SGLT2 (*n* = 69)	-	50.0 (36.0–71.5)	50.0 (38.0–69.5)	0.78
	GLP1 (*n* = 75)	-	45.0 (33.0–66.0)	47.0 (33.0–69.0)	0.60
	Pramlintide (*n* = 32)	-	48.5 (31.5–74.8)	61.0 (35.8–71.0)	1.0
	Colesevelam (*n* = 17)	-	53.0 (32.0–72.0)	48.0 (30.0–71.5)	0.18

Medication classes were included when the sample exceeded 10 patients. ^a^ Indicates statistically significant difference between 2010–2012 and 2015–2016 (*p* < 0.017). ^b^ Indicates statistically significant difference between 2010–2012 and 2016–2017 (*p* < 0.017). ^c^ Indicates statistically significant difference between 2015–2016 and 2016–2017 (*p* < 0.017).

**Table 3:** Results from multivariate linear regression assessing relationship between unique patient characteristics and A1C.

	2015–2016 Study Period			2016–2017 Study Period		
Characteristics	Unstandardized Beta Coefficient *	Standard error	Standardized Beta Coefficient	Unstandardized Beta Coefficient *	Standard error	Standardized Beta Coefficient
Age ^a,c^	−0.002 (−0.002–(−0.002))	<0.001	−0.201	−0.002 (−0.002–(−0.002))	<0.001	−0.195
BMI-Categorized Variable ^b,c^	0.010 (0.003–0.016)	0.003	0.046	0.011 (0.005–0.018)	0.003	0.054
African American/Black ^a,c^	0.088 (0.061–0.114)	0.013	0.106	0.096 (0.070–0.122)	0.013	0.117
Race-Other minority	0.015 (0.061–0.114)	0.011	0.021	0.014 (−0.007–0.036)	0.021	0.021
Female Gender	−0.005 (−0.016–0.006)	0.005	−0.015	−0.007 (−0.018–0.004))	0.005	−0.021
Education Level ^a,c^	−0.010 (−0.014–(−0.006))	0.002	−0.095	−0.012 (−0.015–(−0.008))	0.002	−0.112
Annual Income ^a,c^	−0.008 (−0.012–(−0.005))	0.002	−0.079	−0.007 (−0.011–(0.004))	0.002	−0.073
Adjuvant medication	0.003 (−0.016–0.021)	0.009	0.005	0.010 (−0.008–0.028)	0.009	0.017
Insulin Pump use ^b^	−0.015 (−0.026–(−0.003))	0.006	−0.042	−0.011 (−0.023–0.001)	0.006	0.031
Continuous Glucose Monitor Use ^a,c^	−0.044 (−0.057–(−0.032))	0.006	−0.120	−0.051 (−0.063–(−0.040))	0.006	−0.147

* Parenthesis includes 95% confidence interval of unstandardized beta coefficient. ^a^ *p* value ≤ 0.001 in 2015–2016. ^b^ *p* value ≤ 0.01 and >0.001 in 2015–2016. ^c^ *p* value ≤ 0.001 in 2016–2017.

## Data Availability

Publicly available datasets were analyzed in this study. This data can be accessed at the following link https://public.jaeb.org/datasets/diabetes. Date accessed 24 June 2022.
